# Clinician-ordered peripheral blood smears have low reimbursement and variable clinical value: a three-institution study, with suggestions for operational efficiency

**DOI:** 10.1186/s13000-020-01033-8

**Published:** 2020-09-17

**Authors:** Amy K. Beckman, Valerie L. Ng, David L. Jaye, Manila Gaddh, Sarah A. Williams, Sophia L. Yohe, Lin Zhang, Michael A. Linden

**Affiliations:** 1 Department of Laboratory Medicine and Pathology, University of Minnesota Medical School, Mayo Memorial Building, 420 Delaware Street SE, Minneapolis, MN 55455 USA; 2 Department of Laboratory Medicine & Pathology, Alameda Health System, Oakland, California USA; 3 Department of Laboratory Medicine & Pathology, Emory University Hospital, Atlanta, Georgia USA; 4 Department of Hematology and Medical Oncology, Emory University Hospital, Atlanta, Georgia USA; 5 Division of Biostatistics, University of Minnesota School of Public Health, Minneapolis, MN USA

**Keywords:** Blood film, Peripheral smear, Utilization management, Test utilization

## Abstract

**Background:**

Peripheral blood smears are performed to evaluate a variety of hematologic and non-hematologic disorders. At the authors’ institutions, clinician requests for pathologist-performed blood smear reviews have increased in recent years. Blood smears may contribute significantly to pathologists’ workloads, yet their clinical value is variable, and professional reimbursement rates are low. This study aimed to identify clinical scenarios in which smear review is likely to provide value beyond automated laboratory testing.

**Methods:**

Blood smear review practices at three institutions were examined, and the indications for and interpretations of clinician-initiated smears were reviewed to determine the percentage of smears with potential added clinical value. A smear review was classified as having added clinical value if the pathologist’s interpretation included a morphologic abnormality that had the potential to impact patient management, and that could not be diagnosed by automated complete blood count with white blood cell differential or automated iron studies alone.

**Results:**

Among 515 consecutive clinician-requested smears performed during the study timeframes, 23% yielded interpretations with potential added clinical value. When sorted by indication, 25, 19, and 13% of smear reviews requested for white blood cell abnormalities, red blood cell abnormalities, and platelet abnormalities, respectively, had findings with potential added clinical value. The proportion of smears with potential clinical value differed significantly across these three categories (*p* = 0.0375).

**Conclusions:**

Smear review ordering practices across three institutions resulted in a minority of smears with potential added clinical value. The likelihood of value varied according to the indication for which the smear was requested. Given this, efforts to improve the utilization and efficiency of smear review are worthwhile. Solutions are discussed, including engaging laboratory staff, educating clinicians, and modifying technology systems.

## Background

Peripheral blood smear review is a common test performed to evaluate a wide variety of hematologic and non-hematologic disorders. Evaluation of a peripheral smear by an expert permits assessment of blood cell morphology on a broad level, and may reveal metabolic, nutritional, genetic, and inflammatory abnormalities as well as hemolysis, blood borne parasites, and neoplasia [1]. Smear review is often performed as reflex testing to evaluate abnormalities identified via automated assays such as complete blood count with differential (CBC-D) [2]. Other indications include clinical concern for hemolysis, hematolymphoid neoplasms, and other conditions characterized by abnormalities in blood cell morphology [3]. Despite the proliferation of automated laboratory tests in recent decades, smear review retains its place in the medical armamentarium as an agnostic test with the potential to provide information beyond that detected by automated testing alone [2]. Furthermore, sample procurement poses minimal risk to the patient, and creation of the smear itself is quick and technically straightforward.

Nonetheless, the interpretation of a peripheral blood smear requires time and expertise. Historically, smear review represented a skill practiced by both generalist and specialist physicians. Currently, it is the authors’ impression that smear review is increasingly the domain of pathologists and other laboratory professionals. Most of the thousands of peripheral smear reviews performed annually at the authors’ institutions are interpreted by pathologists, especially hematopathologists. Blood smear review thus constitutes a significant proportion of hematopathologists’ workloads. Despite this, it is not clear how often smear review yields valuable information beyond that provided by automated laboratory tests. Furthermore, pathology departments receive minimal, if any, direct reimbursement for this professional activity. At the University of Minnesota Medical Center (UMMC), for example, there is little reimbursement for the technical component of smears. According to data from 2013 and 2018, the average professional reimbursement was $30, with a range of $0 to $75, depending on the setting and payor. UMMC does not code or bill for a clinical pathology consultation for peripheral smears.

For these reasons, efforts are needed to assess and ultimately improve the utilization of peripheral blood smear review. The goals of this study are to describe blood smear ordering practices and to identify clinical scenarios in which peripheral blood smear review is most likely to be clinically valuable. To accomplish this, we examined the indications for, and results of, clinician-initiated blood smear reviews at three institutions: Highland Hospital (HH), a public hospital located in Oakland, California; Emory University Hospital (EUH), an academic hospital in Atlanta, Georgia; and UMMC, an academic hospital in Minneapolis, Minnesota. Our study offers a broad, multi-institutional perspective on the utilization and value of peripheral blood smear review. We conclude by providing specific suggestions to achieve operational efficiencies to offset an ever-increasing workload.

## Methods

First, the authors outlined the procedures by which peripheral smear reviews were initiated at each institution. Next, peripheral blood smear reviews requested by clinicians and performed consecutively at each institution were identified for the following time periods: HH, from 2009 through 2014; EUH, from December 2015 through April 2016; and UMMC, from December 2015 through January 2016. For each smear, the indication for the review was recorded, along with the final interpretation. Each interpretation was then assessed for potential added clinical value. A smear review was classified as having potential added clinical value if the final interpretation met the following criteria: (1) a morphologic or other diagnostic abnormality was detected by microscopy; (2) the detected abnormality could not be diagnosed by automated CBC-D or automated iron studies alone; and (3) the detected abnormality and overall interpretation were likely to impact patient management. For example, any finding consistent with or suggestive of a hematolymphoid neoplasm or hemoglobinopathy was considered to have potential added clinical value. Morphologic abnormalities (e.g., atypical lymphocytes) that might prompt further evaluation, such as flow cytometry or a bone marrow biopsy, were also classified as potentially valuable. In contrast, interpretations that appeared to be based on history rather than on morphologic features (for example, monoclonal gammopathy of undetermined significance) were not classified as having added clinical value. Smear reviews were then grouped by the indication for which they were requested. The percentage of smears with added clinical value for each indication was calculated, and the results were compared. Finally, statistical analyses using Chi squared tests or Fisher exact tests were performed to assess for differences between the three institutions in terms of the distribution of indications for smear review requests, differences between the institutions in the percentage of smears with potential added clinical value, and differences in rates of potential added clinical value among broad groups of indications.

For the HH dataset, additional medical record review was undertaken for the subset of smears considered to have added clinical value. This was done to determine whether the finding(s) identified on a clinician-ordered smear had been previously reported via the laboratory’s internal review protocol. According to this protocol, CBC-Ds with certain abnormal findings are flagged by laboratory staff and “reflexed” to pathologist review.

## Results

Peripheral blood smear review ordering practices differed among the authors’ three institutions. At HH and UMMC, the majority of blood smears are ordered by clinicians, who request a formal review via their institution’s electronic ordering systems. Attending physicians, residents, fellows, and advanced practice providers from any specialty may request formal smear review by a clinical pathologist/hematopathologist. At both institutions, separate laboratory protocols allow pathologists and laboratory staff to initiate a smear review based on criteria outlined in laboratory procedures, generally related to CBC-D results. These lab-initiated reviews represent a minority of the total number of smears performed by pathologists, and any findings detected via this workflow are not documented in a formal report; instead, they are recorded within the CBC-D results in the electronic medical record. As such, peripheral smear reviews that are the result of internal laboratory protocols are not directly billable.

In contrast, the majority of peripheral blood smear reviews at EUH are initiated and performed by hematologists, without input from pathologists. Only rarely do non-pathologist clinicians request blood smear review by a pathologist. Similar to HH and UMMC, EUH has a separate internal protocol that allows laboratory staff to flag automated CBCs and CBC-Ds for pathologist review without a clinician’s order.

A total of 515 consecutive clinician-requested peripheral blood smear reviews were included in the current study, including 99 from EUH, 216 from HH, and 200 from UMMC. Each smear review was placed into one or more categories according to the indication for which it was initiated (Table 1, Additional file 1). Some smears were ordered for a single reason, e.g., evaluation of hemolysis, and others were requested for more than one reason, e.g., anemia, leukopenia, and thrombocytopenia (pancytopenia). Half of smears were requested to evaluate a red blood cell (RBC) abnormality (*n* = 259; 50%), such as anemia or possible hemolysis. Fewer smears were initiated to evaluate for white blood cell (WBC) abnormalities (*n* = 126, 24%), including leukocytosis, leukopenia, WBC morphology, or blasts; or platelet (PLT) abnormalities (*n* = 150, 29%), for example, thrombocytosis or thrombocytopenia. Smears were commonly ordered to investigate one or more cytopenias (*n* = 267; 52%). Among these, anemia was among the specific indications in 179 (35%), leukopenia in 79 (15%), and thrombocytopenia in 135 (26%). Another frequent reason for smear review was evaluation for hemolysis, which was the indication for 100 smear reviews (19%). Other common indications included suspected or known hematolymphoid neoplasm (65; 13%) and cytosis (53; 10%), particularly leukocytosis (42; 8%). Smaller numbers of smears were requested for assessment of cell (RBC, WBC, or PLT) morphology (39; 8%), or the possible presence of blasts (5; 1%) or parasites (4; 1%). Fifty-five smear reviews (11%) were ordered for a variety of reasons categorized by this study as “other”. These indications included bleeding, thrombosis, coagulation abnormality, fever, weight loss, known or suspected hemoglobinopathy, hemophagocytic lymphohistiocytosis, chronic infection, splenomegaly, lymphadenopathy or mass, unspecified malignancy, liver or kidney failure, or an unspecified abnormality in blood counts or bone marrow biopsy. Finally, no indication was available for 33 smears (6%). The distribution of indications for smear review requests differed significantly among the three institutions (*p* = 4.52 × 10^− 11^), suggesting differences in ordering practices. The majority of this difference lay between EUH versus HH and UMMC, as evidenced by the fact that the *p* value increased when EUH was removed from these calculations (HH versus UMMC, *p* = 4.8 × 10^− 2^; for comparison, EUH versus UMMC, *p* = 3.467 × 10^− 7^; EUH versus HH, *p* = 2.003 × 10^− 12^).

**Table 1:** Indication for and added clinical value of clinician-initiated blood smear reviews

Indication	Smears for this indication, among all smears performed (%)	Smears with added clinical value, among smears performed for this indication (%)
Any	515 (100%)	118 (23%)
Any RBC abnormality	259 (50%)	48 (19%)^a^
Any WBC cell abnormality	126 (24%)	32 (25%)^a^
Any PLT abnormality	150 (29%)	20 (13%)^a^
Any cytosis	53 (10%)	18 (34%)
Erythrocytosis	2 (0%)	0 (0%)
Leukocytosis	42 (8%)	18 (43%)
Thrombocytosis	12 (2%)	1 (8%)
Any cytopenia	267 (52%)	45 (17%)
Anemia	179 (35%)	32 (18%)
Leukopenia	79 (15%)	11 (14%)
Thrombocytopenia	135 (26%)	18 (13%)
Any abnormal cell morphology	39 (8%)	9 (23%)
RBC morphology	22 (4%)	4 (18%)
WBC morphology	6 (1%)	3 (50%)
PLT morphology	5 (1%)	1 (20%)
Hematolymphoid neoplasm	65 (13%)	30 (46%)
Blasts	5 (1%)	2 (40%)
Hemolysis	100 (19%)	24 (24%)
Parasites	4 (1%)	1 (25%)
Other	55 (11%)	15 (27%)
Not specified	33 (6%)	6 (18%)

^a^The proportion of smears with potential clinical value differed significantly across these three categories of indications (*p* = 0.0375)

*RBC* Red blood cell, *WBC* White blood cell, *PLT* Platelet

**Fig. 1:**
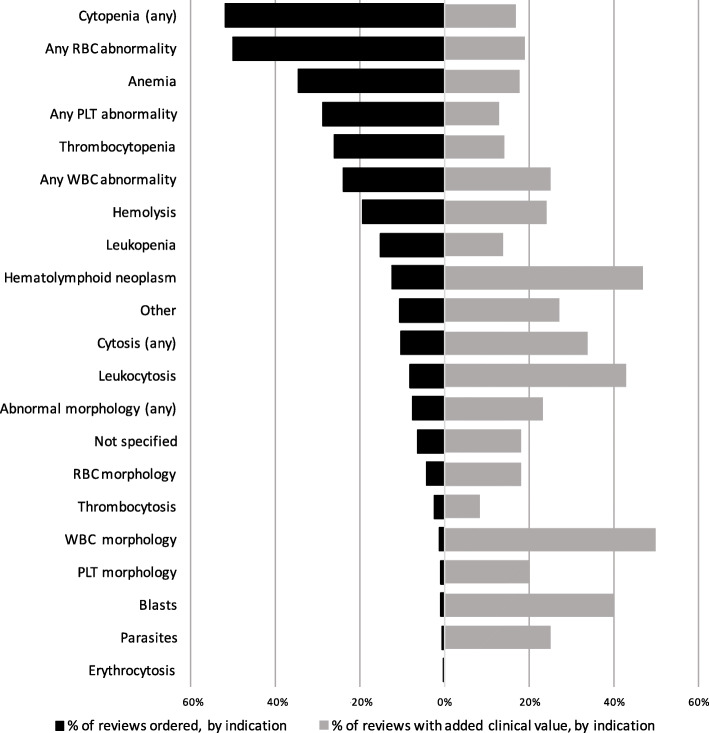
Percentage of clinician-initiated peripheral smear reviews by indication compared to percentage of smear reviews with added clinical value, by indication

One hundred eighteen of 515 (23%) clinician-requested smears yielded potential added clinical value. The percentage of smear reviews with added value varied with the indication for which the smear was performed (Table 1; Fig. 1). Findings with potential added clinical value were seen among 25% of smear reviews requested for reasons related to WBCs, 19% of those requested to assess RBC abnormalities, and 13% of those performed for PLT abnormalities. The proportion of smear reviews with potential clinical value differed significantly across these three categories (*p* = 0.0375). Separate, pairwise calculations comparing the proportion of smear reviews with added value among those requested for WBC, RBC, and PLT abnormalities revealed a significant difference between reviews requested for WBC versus PLT abnormalities (*p* = 0.049). However, there was no significant difference in rates of added clinical value when reviews requested for WBC abnormalities were compared directly with those requested for RBC abnormalities (*p* = 0.309), nor between reviews requested for RBC versus PLT abnormalities (*p* = 0.309).

The specific indication most likely to result in potential added clinical value was evaluation of WBC morphology. Only a small number of smears was requested for this reason (*n* = 6), but half (*n* = 3; 50%) resulted in a potentially valuable interpretation. This fairly high proportion of useful findings was followed closely by smears ordered for assessment of hematolymphoid neoplasm (30 of 65 smears, or 46%, requested for this reason yielded added value), leukocytosis (18 of 42 smears, or 43%, with added value), and assessment for blasts (2 of 5 smears, or 40%, with added value). At the other end of the spectrum, less than 20% of smears performed for any cytopenia; for anemia, leukopenia, or thrombocytopenia specifically; or for RBC morphology, thrombocytosis, or erythrocytosis yielded an interpretation with potential added clinical value.

Interestingly, in some cases, the reason the smear was requested was not directly related to the potentially valuable finding, suggesting the finding was unexpected. These smear reviews represented a subset of all reviews with potential added clinical value; a selection of smears with unexpected findings is listed in Table 2. For example, features consistent with a hemoglobinopathy were found on a review requested to evaluate leukopenia, rouleaux formation was detected on smears performed for assessment of hemolysis, and platelet hypogranularity, but no parasites, was observed on a smear done out of concern for malaria.

**Table 2:** Findings among individual smears requested for select indications

Indication	Findings seen among individual smears requested for the specified indication
Leukocytosis	• Atypical / variant lymphoid cells
	• Findings consistent with a hemoglobinopathy
	• Leukemia or blasts
	• Rouleaux
Anemia	• Atypical/variant lymphoid cells
	• Dysplasia
	• Findings consistent with a hemoglobinopathy
	• Hemolysis
	• Leukemia or blasts
	• Other
	• Platelet abnormality
	• Rouleaux
Leukopenia	• Atypical/variant lymphoid cells
	• Dysplasia
	• Findings consistent with a hemoglobinopathy
	• Hemolysis
	• Leukemia or blasts
	• Platelet abnormality
Thrombocytopenia	• Atypical/variant lymphoid cells
	• Dysplasia
	• Hemolysis
	• Leukemia or blasts
	• Platelet abnormality
	• Rouleaux
Hemolysis	• Dysplasia
	• Findings consistent with a hemoglobinopathy
	• Hemolysis
	• Leukemia or blasts
	• Other
	• Platelet abnormality
	• Rouleaux

Overall, the percentage of smear reviews with added clinical value did not differ among the three institutions (*p* = 0.2002). However, among reviews requested to evaluate patients for hemolysis specifically, a significantly greater percentage of reviews performed at EUH yielded a valuable interpretation than at HH or UMMC (*p* = 0.0007).

For the HH dataset, medical record review was performed for all 57 smears considered to have added clinical value. Of these smears, almost half (*n* = 26; 46%) had been previously referred to a pathologist for review following internal laboratory protocols. These initial pathologist reviews occurred prior to formal clinician requests for blood smear review, and were reported in the patient’s electronic health record as comments attached to recent CBC results.

## Discussion

Our findings confirm that peripheral blood smear review practices differ among institutions, and that smear reviews are requested by clinicians for a wide variety of indications. These results are drawn from a large number of smear reviews performed across three institutions. Overall, we found that less than one-quarter of clinician-initiated smear reviews yielded positive findings with potential clinical value beyond that provided by automated iron studies alone. Not surprisingly, the likelihood of a potentially valuable interpretation varied according to the reason the review was performed. Among smears requested for reasons related to WBC abnormalities, 25% yielded findings with potential added clinical value, compared to 19% of smears requested to assess RBC abnormalities and 13% of smears performed for PLT abnormalities. Notably, the indications with the highest rates of potential clinical value (WBC number, WBC morphology, and assessment for hematolymphoid malignancy) were infrequent. In contrast, the most common indications for clinician-requested smear review (anemia and thrombocytopenia; 35 and 26% of indications, respectively) were less likely to yield added value, with just 18 and 13% of reviews yielding potentially valuable interpretations.

Peripheral blood smear review is broadly accepted as a key step in the workup of many clinical issues [2, 3]. However, the test predates the automated hematology and chemistry assays that are now commonplace in medical centers in resource-rich areas. Modern hematology analyzers, for example, measure several RBC parameters, such as mean corpuscular volume, mean corpuscular hemoglobin concentration, and red cell distribution width, and can detect platelet clumping. These parameters provide an assessment of RBC morphology and may identify pseudothrombocytopenia, in some cases obviating the need for manual review.

In cases where manual review is performed, data supporting its value are sparse. A handful of studies have questioned the contribution of smear review to medical management (Table 3). Studies examining the role of smear review in the evaluation of anemia specifically found that peripheral blood smear was unreliable for the diagnosis of iron deficiency anemia [4], seldom made a novel contribution to the care of anemic inpatients [5], and failed to positively impact diagnostic accuracy or laboratory workup of anemia [6]. In a study involving peripheral smears ordered by clinicians for a variety of indications, Kurt-Mangold et al. [7] reported that only 1% of pathologist-performed blood smear reviews provided unique data with a clear impact on patient management. This very low rate of clinical value contrasts with our study’s findings, a discrepancy that is likely the result of the way in which the authors defined clinical value. Kurt-Mangold and colleagues required medical record documentation of the effect of smear review on clinical decision-making [7], which may have underestimated the clinical value of smear review. At the same time, our study may have overestimated the clinical impact of blood smear review, because we relied on the presence of morphologic abnormalities on the smear, rather than on evidence of impact on patient management.

**Table 3:** Studies examining the clinical value of peripheral blood smear review

Author, publication date, and institution	Study subjects and design	Results	Authors’ conclusions
Fairbanks et al. [4] 1971 Mayo Clinic, Rochester, Minnesota	2 blood smears per subject (24 normal controls + 38 patients with IDA) reviewed, for the purposes of the study, by 9 staff and resident hematologists	Among all interpretations: • False positives (IDA reported on a control patient): 5.8% • False negatives (IDA not reported on IDA case): 51.0% • Consensus among all 9 reviewers: 5 of 38 blood smears from IDA patients Average intra observer variability (discrepancy when a reviewer reviewed the same smear twice): 22%	“Except when morphologic changes are pronounced, the diagnosis of iron deficiency anemia from examination of the peripheral blood film is difficult and not very reliable.”
Jen et al. [5] 1983 Brigham and Women’s Hospital, Boston, Massachusetts	288 anemic inpatients with blood smears reviewed in the context of clinical care by laboratory staff, with or without physician review	• Only 5 of 11 (45%) common RBC morphologic abnormalities showed both inter- and intra-observer reproducibility better than chance. • Among patients evaluated for iron, folate, or B12 deficiency, red cell indices showed similar or better specificity and positive predictive value than blood smear interpretation. • Among smears interpreted by laboratory staff, additional physician interpretation yielded “unique” diagnostic information in no cases and “helpful” information in 2.2% of cases.	• “Blood smear readings are poorly reproducible, are no better than RBC indices for screening for possible deficiency states, and only occasionally provide unique information.” • “The physician’s reading is most important for the confirmation of abnormal WBC morphology and is most likely to add incremental value in patients whose elevated reticulocyte counts suggest hemolysis.”
Simmons et al. [6] 1989 Walter Reed Army Medical Center, Washington, DC	12 cases of anemia with blood smear and clinical and CBC data reviewed, for the purposes of the study, by 65 residents, fellows, and staff physicians	• Access to a peripheral smear did not significantly change the number or appropriateness of tests ordered. • Access to a peripheral smear did not significantly improve the reviewer’s ability to make a correct diagnosis.	“RBC review, even when accurate, does not improve clinical problem solving across a variety of common anemias and among a broad cross section of residents, internists, and hematologists.”
Kurt-Mangold et al. [7] 2018 University of Iowa Hospitals and Clinics	Chart review of patients associated with 277 clinician-ordered peripheral blood smear reviews	• 68% of smear review reports included unique data beyond that already described in the medical record. • 52% of smear review results were not mentioned in patients’ clinical notes. • Data obtained from peripheral smear review impacted clinical decision making in 1% of cases.	“Only rarely do [data from peripheral blood smear review] appear to be clinically significant and the information frequently overlaps with information already provided by laboratory-initiated smear reviews.”

*CBC* Complete blood count, *IDA* Iron deficiency anemia, *RBC* Red blood cell

The manner in which we defined clinical utility represents a limitation of our study. In addition, our study did not recognize situations in which a morphologic finding detected on peripheral smear represented a previously established diagnosis. Furthermore, we considered only positive findings as potentially valuable, when in actuality, negative results are often critical to the formation of a differential diagnosis. However, serious hematologic disorders are typically associated with CBC abnormalities. As such, they are likely to be captured by internal laboratory protocols, as evidenced by our subset analysis of the HH dataset. This analysis demonstrated that at least some of the smear reviews requested by clinicians were redundant, having already been performed as a result of laboratory protocols. In such cases, a comment was added to the CBC results in the patient’s electronic heath record. The redundant requests were likely related to viewers not recognizing the presence or significance of such a comment, or to a lack of adequate communication with the clinical care team. It is also possible that a comment attached to the CBC-D is seen as a preliminary result, prompting the clinician to request formal blood smear review. An additional limitation of our study is that our data were insufficient to compare the rate of potential added clinical value of smear reviews ordered by clinicians versus reviews initiated by laboratory staff.

The increased demand for pathologist peripheral smear reviews may be an indicator of a previously unmet clinical need. The true value of pathologist-performed blood smear review may lie not with the morphologic findings detected, but rather with the clinical consultation rendered by the pathologist. The HH dataset, for example, included cases for which the pathologist synthesized a final diagnosis (e.g., sideroblastic anemia, thalassemia, cytopenias most likely related to underlying cirrhosis) by integrating existing laboratory and clinical information. Surprisingly, a number of peripheral blood smear reviews, categorized by the current study as having potential added clinical value, were requested for morphological verification of sickle cell anemia, information used by the clinician to distinguish sickle cell pain crisis from drug seeking behavior. In other cases, smear reviews were requested to screen for hematologic disorders better diagnosed by other methods, such as hairy cell leukemia, lymphoma, and filariasis. Clearly, pathologists possess fundamental knowledge regarding the use of laboratory data to diagnose hematologic disorders, and can add value to patient care in a general medical practice setting.

Peripheral smear review represents a labor-intensive test that may have limited value when broadly applied. Consequently, its use should be limited to scenarios where it is likely to make a unique contribution to patient care. To this end, we propose that institutions examine their peripheral blood smear review practices, recognizing that smear review is most likely to add value when requested to evaluate WBC abnormalities. Education of clinicians who order this test might improve diagnosis of common hematologic disorders based on existing clinical and laboratory reviews, while limiting the number of reviews that are unlikely to provide uniquely valuable information. Laboratory test utilization practices are often propagated at morbidity and mortality and morning report conferences. Such conferences may represent a good arena for education and representation by pathologists.

We also suggest that laboratory staff be engaged in the screening and triaging of clinician-ordered peripheral smear reviews. Rules guiding laboratory response to abnormalities observed on automated CBC-D are common [2, 8–10]; according to such protocols, a CBC that meets certain pre-defined parameters undergoes reflex morphologic review by a technologist, who then determines whether additional review by a pathologist is warranted. Similar criteria could be applied to clinician-initiated smear reviews, thereby limiting pathologist review to specimens most likely to benefit from their expertise. At one of the authors’ institutions (UMMC), a medical technologist serves as a physician extender, drafting reports for a majority of clinician-ordered peripheral blood smears prior to pathologist review. Other institutions outside of this study have procedures that guide interpretative comments. For cases that undergo reflex pathologist review, improvements in communication among clinical teams, along with better portrayal of findings in the electronic health record, may prevent redundant smear review.

Finally, technological improvements may improve the efficiency of peripheral smear review. At UMMC, for example, the laboratory information system was amended to automatically integrate CBC-D data into peripheral blood smear review reports, thereby saving pathologist time, reducing transcription errors, and ensuring the correct reference ranges are added. To reduce unnecessary repeat reviews, our technologists contact providers who order repeat reviews on a single patient within 7 days. Because these repeat orders are often canceled, this step has reduced the number of blood smear reviews by about 5–8% (unpublished data). We plan to automate this practice by modifying the computerized provider order entry system with a “soft stop” that provides a dialogue box when more than one smear is requested per week, encouraging clinicians to consult with a hematopathologist. In the future, we anticipate that laboratories may employ algorithms, perhaps developed using artificial intelligence, to integrate patient characteristics with automated test results, in order to identify patients most likely to benefit from peripheral blood smear review.

## Conclusions

In summary, peripheral blood smear review is a labor-intensive, poorly reimbursed study whose broad application shows variable clinical value. According to this multi-institutional study, smear review ordering practices vary, but overall result in a minority of smear reviews with potential added clinical value beyond automated laboratory testing alone. When sorted according to the general reason they were requested, 25% of smears requested for WBC abnormalities, 19% of smears ordered for RBC abnormalities, and 13% of smears initiated for PLT abnormalities had findings with potential added clinical value. The indications with the highest rates of potential clinical value (WBC number, WBC morphology, and assessment for hematolymphoid malignancy) were infrequent. In contrast, smears requested to evaluate anemia and thrombocytopenia, the most common reasons for peripheral smear review requests, had lower rates of potential clinical value, aside from confirming the absence of significant findings. To improve the utilization and efficiency of peripheral blood smear review, we propose that laboratory staff participate in screening and triage, that clinicians and pathologists engage in discussion regarding clinical scenarios most likely to benefit from smear review, and that laboratory information and computerized provider order entry systems be modified to support pathologists.

## Supplementary information

**Additional file 1.** Indications for and added clinical value of clinician-initiated peripheral blood smear reviews among three institutions.

## Data Availability

The datasets generated and analyzed during the current study are available from the corresponding author on reasonable request.

## Abbreviations

CBC: Complete blood count
CBC-D: Complete blood count with white blood cell differential
EUH: Emory University Hospital
HH: Highland Hospital
PLT: Platelet
RBC: Red blood cell
UMMC: University of Minnesota Medical Center
WBC: White blood cell
