# Supplementary material for: Clinician-ordered peripheral blood smears have low reimbursement and variable clinical value: a three-institution study, with suggestions for operational efficiency
Source: Diagn Pathol. 2020 Sep 17;15:112. doi: 10.1186/s13000-020-01033-8 (PMC7500005; doi:10.1186/s13000-020-01033-8)
Supplement: Supplementary file 1 — Additional file 1. Indications for and added clinical value of clinician-initiated peripheral blood smear reviews among three institutions. [file 13000_2020_1033_MOESM1_ESM.pdf]

Additional file 1. Indications for and added clinical value of clinician-initiated peripheral blood smear reviews among three institutions

	Total (n=515)		Emory University Hospital (n=99)		Highland Hospital (n=216)		University of Minnesota Medical Center (n=200)	
**Indication**	**Smears for this indication (%)***	**Smears with added clinical value (%)****	**Smears for this indication (%)***	**Smears with added clinical value (%)****	**Smears for this indication (%)***	**Smears with added clinical value (%)****	**Smears for this indication (%)***	**Smears with added clinical value (%)****
**Any**	515 (100%)	118 (23%)	99 (100%)	23 (23%)	216 (100%)	57 (26%)	200 (100%)	38 (19%)
**Any RBC abnormality**	259 (50%)	48 (19%)	43 (43%)	6 (14%)	109 (50%)	23 (21%)	107 (54%)	19 (18%)
**Any WBC abnormality**	126 (24%)	32 (25%)	24 (24%)	4 (17%)	63 (29%)	17 (27%)	39 (20%)	11 (28%)
**Any PLT abnormality**	150 (29%)	20 (13%)	47 (47%)	5 (11%)	54 (25%)	9 (17%)	49 (25%)	6 (12%)
**Any cytosis**	53 (10%)	18 (34%)	6 (6%)	2 (33%)	32 (15%)	12 (38%)	15 (8%)	4 (27%)
**Erythrocytosis**	2 (0%)	0 (0%)	0 (0%)	0 (0%)	1 (0.5%)	0 (0%)	1 (0.5%)	0 (0%)
**Leukocytosis**	42 (8%)	18 (43%)	3 (3%)	2 (67%)	29 (13%)	12 (41%)	10 (5%)	4 (40%)
**Thrombocytosis**	12 (2%)	1 (8%)	4 (4%)	1 (25%)	4 (2%)	0 (0%)	4 (2%)	0 (0%)
**Any cytopenia**	267 (52%)	45 (17%)	69 (70%)	7 (10%)	89 (41%)	19 (21%)	109 (55%)	19 (17%)
**Anemia**	179 (35%)	32 (18%)	40 (40%)	3 (8%)	64 (30%)	15 (23%)	75 (38%)	14 (19%)
**Leukopenia**	79 (15%)	11 (14%)	19 (19%)	0 (0%)	32 (15%)	5 (16%)	28 (14%)	6 (21%)
**Thrombocytopenia**	135 (26%)	18 (13%)	44 (44%)	4 (9%)	48 (22%)	8 (17%)	43 (22%)	6 (14%)
**Any abnormal cell morphology**	39 (8%)	9 (23%)	1 (1%)†	1 (100%)	26 (12%)	6 (23%)	12 (6%)	2 (17%)
**RBC morphology**	22 (4%)	4 (18%)	0 (0%)	0 (0%)	17 (8%)	3 (18%)	5 (3%)	1 (20%)
**WBC morphology**	6 (1%)	3 (50%)	0 (0%)	0 (0%)	4 (2%)	2 (50%)	2 (1%)	1 (50%)
**PLT morphology**	5 (1%)	1 (20%)	0 (0%)	0 (0%)	3 (1%)	1 (33%)	2 (1%)	0 (0%)
**Hematolymphoid neoplasm**	65 (13%)	30 (46%)	20 (20%)	10 (50%)	23 (11%)	12 (52%)	22 (11%)	8 (36%)
**Blasts**	5 (1%)	2 (40%)	0 (0%)	0 (0%)	5 (2%)	2 (40%)	0 (0%)	0 (0%)
**Hemolysis**	100 (19%)	24 (24%)	5 (5%)	5 (100%)	53 (25%)	12 (23%)	42 (21%)	7 (17%)
**Parasites**	4 (1%)	1 (25%)	0 (0%)	0 (0%)	2 (1%)	1 (50%)	2 (1%)	0 (0%)
**Other**	55 (11%)	15 (27%)	4 (4%)	0 (0%)	23 (11%)	11 (48%)	28 (14%)	4 (14%)
**Not specified**	33 (6%)	6 (18%)	0 (0%)	0 (0%)	28 (13%)	6 (21%)	5 (3%)	0 (0%)

*Percentages represent the proportion of peripheral smear reviews performed for a given indication, among all smear reviews at the specified institution(s). Many smear reviews were initiated for more than one indication. As a result, the sum of the percentages in these columns is greater than 100%.
**Percentages represent the proportion of smear reviews with added clinical value, among all smear reviews performed for this indication at the specified institution(s).
†This smear was requested to evaluate cell morphology; however, no specific lineage was specified.
RBC, red blood cell; WBC, white blood cell; PLT, platelet
